# How multidisciplinary clinics may mitigate socioeconomic barriers to care for chronic limb-threatening ischemia

**DOI:** 10.1016/j.jvs.2024.05.033

**Published:** 2024-06-19

**Authors:** Drayson B. Campbell, Goutam Gutta, Carly G. Sobol, Said A. Atway, Mounir J. Haurani, Xiaodong P. Chen, Vincent L. Rowe, Mitchel R. Stacy, Michael R. Go

**Affiliations:** a Ohio State University College of Medicine, Columbus; b Division of Vascular Diseases and Surgery, Department of Surgery, The Ohio State University Wexner Medical Center, Columbus; c Division of Vascular Surgery, Department of Surgery, University of Wisconsin, Madison; d Department of Orthopaedics, The Ohio State University College of Medicine, Columbus; e Department of Surgery, The Ohio State University Wexner Medical Center, Columbus; f David Geffen School of Medicine, UCLA, Los Angeles; g Center for Regenerative Medicine, The Research Institute at Nationwide Children’s Hospital, Columbus

**Keywords:** CLTI, Multidisciplinary, Socioeconomic

## Abstract

**Objective:**

Although multidisciplinary clinics improve outcomes in chronic limb-threatening ischemia (CLTI), their role in addressing socioeconomic disparities is unknown. Our institution treats patients with CLTI at both traditional general vascular clinics and a multidisciplinary Limb Preservation Program (LPP). The LPP is in a minority community, providing expedited care at a single facility by a consistent team. We compared outcomes within the LPP with our institution’s traditional clinics and explored patients’ perspectives on barriers to care to evaluate if the LPP might address them.

**Methods:**

All patients undergoing index revascularization for CLTI from 2014 to 2023 at our institution were stratified by clinic type (LPP or traditional). We collected clinical and socioeconomic variables, including Area Deprivation Index (ADI). Patient characteristics were compared using *χ*^2^, Student *t*, or Mood median tests. Outcomes were compared using log-rank and multivariable Cox analysis. We also conducted semi-structured interviews to understand patient-perceived barriers.

**Results:**

From 2014 to 2023, 983 limbs from 871 patients were revascularized; 19.5% of limbs were treated within the LPP. Compared with traditional clinic patients, more LPP patients were non-White (43.75% vs 27.43%; *P* < .0001), diabetic (82.29% vs 61.19%; *P* < .0001), dialysis-dependent (29.17% vs 13.40%; *P* < .0001), had ADI in the most deprived decile (29.38% vs 19.54%; *P* = .0061), resided closer to clinic (median 6.73 vs 28.84 miles; *P* = .0120), and had worse Wound, Ischemia, and foot Infection (WIfI) stage (*P* < .001). There were no differences in freedom from death, major adverse limb event (MALE), or patency loss. Within the most deprived subgroup (ADI >90), traditional clinic patients had earlier patency loss (*P* = .0108) compared with LPP patients. Multivariable analysis of the entire cohort demonstrated that increasing age, heart failure, dialysis, chronic obstructive pulmonary disease, and increasing WIfI stage were independently associated with earlier death, and male sex was associated with earlier MALE. Ten traditional clinic patients were interviewed via convenience sampling. Emerging themes included difficulty understanding their disease, high visit frequency, transportation barriers, distrust of the health care system, and patient-physician racial discordance.

**Conclusions:**

LPP patients had worse comorbidities and socioeconomic deprivation yet had similar outcomes to healthier, less deprived non-LPP patients. The multidisciplinary clinic’s structure addresses several patient-perceived barriers. Its proximity to disadvantaged patients and ability to conduct multiple appointments at a single visit may address transportation and visit frequency barriers, and the consistent team may facilitate patient education and improve trust. Including these elements in a multidisciplinary clinic and locating it in an area of need may mitigate some negative impacts of socioeconomic deprivation on CLTI outcomes.

Chronic limb-threatening ischemia (CLTI) is the most severe form of peripheral artery disease and has become increasingly prevalent due to the aging population, rising obesity rates, and the diabetes mellitus (DM) epidemic.^1–3^ Patients with CLTI often suffer from numerous comorbidities, require intensive care from multiple specialties, and have high rates of mortality, adverse cardiovascular events, and amputations.^1,2,4–9^ Furthermore, significant disparities in CLTI have been documented, and patients with CLTI with poor health care access, low health care literacy, minority status, and high levels of socioeconomic distress suffer disproportionately compared with less disadvantaged patients.^1,2,10–16^

Meanwhile, multidisciplinary limb salvage centers are associated with improved outcomes in CLTI compared with traditional models of separate specialty-specific care, although the specific mechanisms behind this effect remain unclear.^17–22^ There has not yet been an investigation into the role multidisciplinary clinics may have in reducing disparities within CLTI.

We sought to investigate the intersection of multidisciplinary clinics and disparities within CLTI. Our institution treats patients with CLTI at both traditional general vascular specialty-specific clinics and at a multidisciplinary Limb Preservation Program (LPP). The LPP is purposefully located in a minority community with high rates of renal failure, DM, and CLTI and provides expedited inpatient and outpatient care at a single facility by a consistent team of a vascular surgeon, physician’s assistant, and podiatrist within a wound center.^23^ In addition to investigating outcome differences between traditional and multidisciplinary clinics, we sought to explore the ways by which multidisciplinary clinics such as the LPP may impact barriers to care experienced by patients. We hypothesized that superior outcomes within multidisciplinary clinics may result from improved access to care and may particularly benefit socioeconomically deprived patients.

## METHODS

### Institutional information.

After approval from an Institutional Review Board (The Ohio State University Biomedical Sciences Institutional Review Board protocol number 2019H0219), we conducted an explanatory sequential mixed-methods study including quantitative and qualitative components. The quantitative part was a retrospective cohort study designed to examine CLTI outcomes of patients who were treated at our institution’s multidisciplinary LPP compared with those treated at any of our institution’s traditional specialty-specific clinics. The qualitative part was designed to explore patients’ perspectives regarding perceived barriers they experienced while receiving care.

From 2014 to 2023, our institution had eight traditional general vascular clinics that provided care to patients with CLTI. At any given time, approximately six vascular surgeons and one interventional cardiologist were providing CLTI care at these sites. After intervention, typically patients are seen every 6 months for clinical evaluation and noninvasive arterial assessment with duplex ultrasonography and ankle-brachial indices. Other follow-up such as specialty-specific wound care or podiatry consultation is at the discretion of the surgeon.

Separately, the institution’s LPP is located within a satellite hospital situated in a socioeconomically deprived community. The LPP is a multidisciplinary service where assessment, testing, and rendering of multispecialty care occurs at a single location as efficiently as possible.^23^ Housed within the institution’s wound center, the LPP holds one outpatient clinic day per week, with referrals coming from wound center providers, community podiatrists, outlying wound centers, and referrals following hospitalizations. At an initial visit, patients receive vascular and podiatric evaluation, debridement, arterial and venous testing, on-site prosthetic and orthotic evaluation, and scheduling of coordinated treatment.

The LPP has 12 outpatient visit rooms, two hyperbaric chambers, and a dedicated clinical research space. The adjacent, physically connected hospital contains catheterization laboratories, operating rooms, and a dedicated inpatient unit. A single vascular surgeon (who also saw patients at several traditional general vascular clinics) and a physician assistant (PA) ran the LPP outpatient and inpatient services.

Protocols ensure consistent structured diagnostic approaches, testing, and vascular and wound care follow-up.^24^ Follow-up occurs every 2 to 4 weeks for wound care, where evaluation by the vascular surgeon, PA, and podiatrist occurs, along with additional specialists (eg, plastic or orthopedic surgeons) as needed. Noninvasive arterial assessment occurs every 6 months. Biweekly multidisciplinary conferences are held to discuss complex cases. The program also provides clinical training for residents and students in vascular surgery, general surgery, and podiatry.

### Quantitative methods.

A retrospective cohort study was conducted among all patients who received index endovascular intervention or surgical bypass for CLTI at our institution from January 2014 to March 2023.

Patient information was collected according to Society for Vascular Surgery reporting standards.^25^ Wound, Infection, and Ischemia (WIfI) stage, TransAtlantic Inter-Society Consensus (TASC) classification, level of disease, and location of treatment (LPP vs traditional clinic) were collected.^26,27^ For limbs with multiple disease levels, all TASC classes were collected, and only the most severe was considered. Socioeconomic data included distance from clinic, residence in a nursing home, need for an interpreter, and Area Deprivation Index (ADI).^28,29^ The ADI ranks neighborhoods (Census block groups) in the United States based on factors for the domains of income, education, employment, and housing quality; a higher score indicates worse deprivation. Given the ADI is most useful in informing health delivery and policy among the most disadvantaged groups, we added a classification for patients in the most disadvantaged decile (ADI >90) to identify patients with the greatest deprivation.

Primary outcomes included freedom from death, major adverse limb event (MALE), and patency loss. MALE was defined as above-ankle amputation or reintervention in the affected limb. Loss of patency was defined as a loss of a previously palpable pedal pulse, a velocity ratio >2.5 on arterial duplex, a drop in ankle-brachial index of >0.15, or when repeat intervention was performed in the target arterial path.^23,25,30,31^

Two-sample independent *t*-tests were used to compare continuous variables (shown as mean ± standard deviation). Continuous variables that did not pass the Shapiro-Wilk test for normality were compared using Mood median tests (shown as median [interquartile range (IQR)]). *χ*^2^ or Fisher exact tests were used for categorical variables (shown as category counts and percentages). Product-limit Kaplan-Meier was used to estimate survival functions, with curves truncated at standard errors of 10%. Between-group comparisons were also made using univariable Cox proportional hazards analyses. Multivariable analyses were then conducted to determine hazard ratios (HRs) for each outcome. These analyses included six preselected variables (clinic type, index intervention type, age, race, sex, and ADI >90) along with all variables that had a *P* value less than .20 on the relevant univariable Cox proportional hazards analysis. For the proportional hazards analyses, WIfI stage and TASC were treated as ordinal variables such that HRs indicate risk for each 1-unit incremental increase. All multivariable models were ensured to have greater than 10 events per variable. All analyses were performed at an alpha level of 0.05 using R Studio (version 4.2.2).

### Qualitative methods.

The qualitative component was designed to understand patient perspectives on barriers to care via semi-structured interviews. Eligible participants were those who received any intervention for CLTI within the last 6 months. Convenience sampling was used; eligible participants were invited to participate in 20- to 40-minute phone-based interviews. Following consent, participants were asked about their experience with three primary domains for CLTI care: initial referral, vascular care, and wound care. Open-ended questions were used to elicit general experiences (eg, “How were you first connected to vascular surgery?”, “Can you tell me about the care you received for any wounds on your legs?”). Patients were then specifically asked about challenges/barriers they experienced (eg, “What challenges did you have in continuing your care with our vascular surgery team?”). Patient-identified challenges/barriers were then iteratively explored to obtain in-depth perspectives on the origin of the barrier and any participant-perceived remedies. Interviewers avoided leading or closed-ended questions about potential challenges or solutions, and themes from past interviews were not introduced by the interviewer. This process was repeated for each domain for all interviewed participants.

After interview transcription and de-identification, analysis was performed using the Framework Method.^32^ We adopted an inductive approach to thematic analysis; thematic categories were not predetermined. Instead, categories were developed through iterative discussion of patient quotes that authors coded from transcripts. Three authors (DBC, GG, MRG) met after every two to three interviews to review these quotes. The cumulative array of coded quotes was then used to organize discussion of commonalities, termed “thematic categories.” After every series of interviews, these categories were adapted and reclassified until consensus was reached. Participant recruitment was determined complete once data saturation was reached and thematic occurrence stabilized.

## RESULTS

### Quantitative results.

Between January 2014 and March 2023, 983 limbs from 871 patients received successful index revascularization for CLTI. Of these limbs, 192 (19.5%) received treatment at the multidisciplinary LPP. Over the course of the study period, 332 patients died, and 383 limbs had a MALE.

Patient characteristics were compared between limbs treated at the multidisciplinary LPP vs traditional clinics (Table I). Of note, ADI and distance distributions were non-normal (Shapiro-Wilk normality test W = 0.945 and *P* < .0001 and W = 0.455 and *P* < .0001, respectively). Patients treated at the LPP were more often non-White (43.75% vs 27.43%; *P* < .0001), diabetic (82.29% vs 61.19%; *P* < .0001), dialysis-dependent (29.17% vs 13.40%; *P* < .0001), residents of a nursing home (13.54% vs 7.84%; *P* = .0192), not prescribed a postoperative P2Y12 inhibitor (63.54% vs 71.70%; *P* = .0335), and not prescribed a postoperative statin (73.44% vs 80.96%; *P* = .0267). LPP patients resided closer to clinic (6.73 [IQR, 3.83-23.34] miles vs 28.84 [IQR, 8.42-54.52] miles; *P* = .0120), had a more left-skewed ADI distribution (71 [IQR, 56-92] vs 72 [IQR, 54-88]; *P* < .0001), and were more often in the most deprived ADI decile (29.38% vs 19.54%; *P* = .0061). Additionally, fewer LPP patients had aortoiliac disease (12.50% vs 26.04%; *P* = .0001), but more had tibial disease (44.79% vs 34.01%; *P* = .0068). A greater proportion of LPP patients received index endovascular intervention rather than open bypass (81.77% vs 71.68%; *P* = .0059), and LPP patients had significantly worse WIfI stage (*P* < .0001). Clinic strata did not differ when grouped by other collected characteristics.

**Fig 1.**
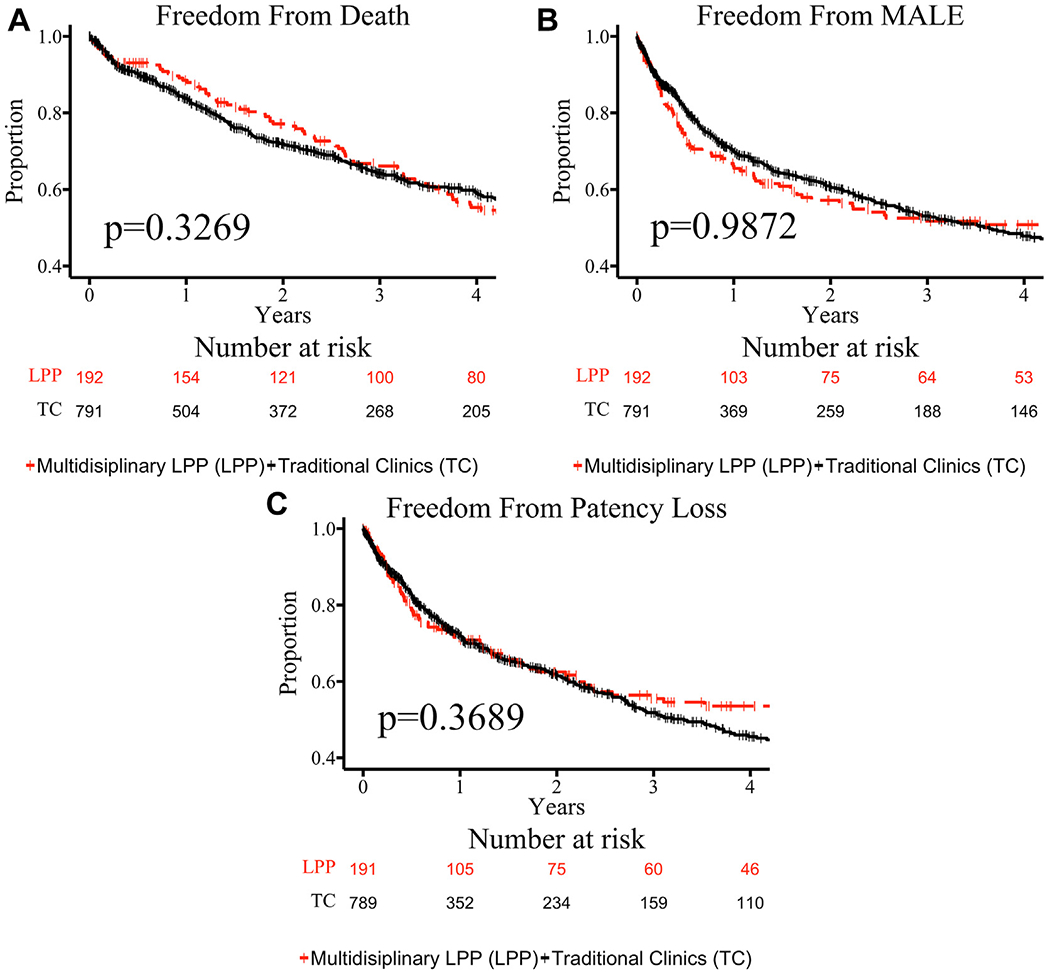
Kaplan-Meier plots are shown for the primary outcomes of the entire cohort of patients who received index revascularization: death **(A)**, major adverse limb event (*MALE*) **(B)**, and loss of patency **(C)**. Functions are grouped by the clinic type at which they received treatment: multidisciplinary Limb Preservation Program (*LPP*) shown in *dashed red* and traditional clinics (*TC*) shown in *solid black*. Censored values are shown with “|.” Standard errors of curves do not exceed 10% for the time frames shown. Functions did not differ between groups for any outcome shown.

**Fig 2.**
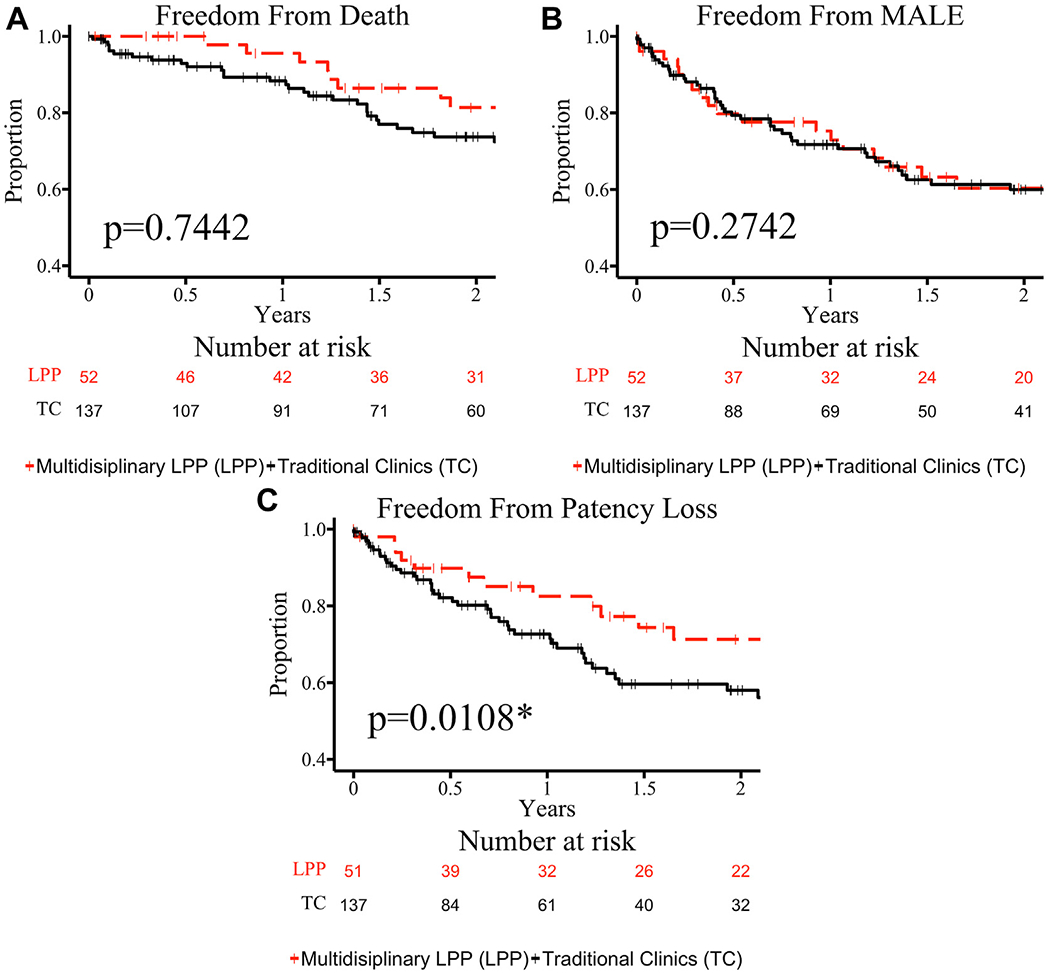
Kaplan-Meier plots are shown for the primary outcomes of patients in the most deprived Area Deprivation Index (ADI) decile (ADI >90) who received index revascularization: death **(A)**, major adverse limb event (*MALE*) **(B)**, and loss of patency **(C)**. Functions are grouped by the clinic type at which they received treatment: multidisciplinary Limb Preservation Program (*LPP*) shown in *dashed red* and traditional clinics (*TC*) shown in *solid black*. Censored values are shown with “|.” Standard errors of curves do not exceed 10% for the time frames shown. Functions differed between groups for patency loss (*P* = .0108, noted with a “*”) but not for death and MALE.

Time-dependent outcomes after index intervention were compared between clinic strata (Fig 1). Freedom from death, MALE, and loss of patency did not differ between clinic strata. Time-dependent outcomes after index intervention in the most deprived patients (ADI >90) were then compared between clinic strata (Fig 2). Freedom from death and MALE did not differ between the ADI >90 clinic strata. However, freedom from loss of patency was greater in the LPP ADI >90 group compared with the traditional clinic ADI >90 group (log-rank *P* = .0108).

Within the entire cohort, multivariable analysis of freedom from death (Table II) identified increasing age (HR, 1.03; 95% confidence interval [CI],1.02-1.04; *P* < .001), congestive heart failure (1.96; 95% CI, 1.48-2.61; *P* < .001), dialysis dependence (2.22; 95% CI, 1.62-3.03; *P* < .001), chronic obstructive pulmonary disease (1.47; 95% CI, 1.14-1.90; *P* = .003), and worsening WIfI stage (1.13; 95% CI, 1.01-1.27; *P* = .045) as independent risk factors for earlier death. Multivariable analysis of freedom from MALE (Table III) identified male sex (1.42; 95% CI, 1.11-1.82; *P* = .006) as the only independent risk factor for earlier MALE.

Multivariable analysis of freedom from loss of patency (Supplementary Table I, online only) identified dialysis dependence (1.57; 95% CI, 1.15-2.14; *P* = .005) as an independent risk factor for earlier loss of patency.

Among patients with an ADI >90, multivariable analysis identified treatment at a traditional clinic (2.17; 95% CI, 1.08-4.38; *P* = .030) as an independent risk factor for earlier loss of patency while controlling for age, sex, race, smoking history, aortoiliac disease, tibial disease, multilevel disease, and index intervention. Otherwise, clinic type did not emerge as a significant variable in multivariable analyses of any other primary endpoint within the entire cohort or the ADI >90 subgroup (data not shown).

### Qualitative results.

In the qualitative portion, 10 participants were interviewed, all of whom were followed at traditional clinics. Among the 10 interviewed participants, the average age was 67.4 ± 9.4 years, two participants (20%) were Black, and seven participants (70%) were male. Four participants (40%) had Medicaid or paid out of pocket, whereas six (60%) had commercial, military, or Medicare insurance. The median ADI was 67 (IQR, 60-89), and two (20%) participants had an ADI in the worst decile (ADI >90).

Five themes of patient-perceived barriers emerged from interviews: difficulty understanding their disease, high visit frequency, transportation barriers, distrust of the health system, and patient-physician racial discordance. Illustrative phrases for each theme are shown (Table IV), and the complete list of indexed phrases is available (Supplementary Table II, online only). Transportation was the most reported barrier, resulting from either a lack of vehicle access or medical disability prohibiting driving. Difficulty understanding disease stemmed from a gap in understanding disease severity or appropriate treatment modalities. High visit frequency occurred in patients with numerous non-CLTI comorbidities requiring separate appointments or multiple dyssynchronous CLTI-related appointments. Distrust of the health system was demonstrated both regarding the vascular surgeon treating their condition and the health system as a whole. Lastly, patient-physician racial discordance presented challenges for patients in understanding accents of physicians and other health care staff.

## DISCUSSION

In our study, compared with patients treated at traditional clinics, patients treated within the multidisciplinary LPP were more likely to be a racial minority, be more socioeconomically deprived, have DM, have dialysis-dependent renal failure, have less optimized medical management, reside in a nursing home, have limbs with worse WIfI stage, and receive index endovascular intervention as opposed to bypass. Within the literature, many of these characteristics have been shown to be associated with worse CLTI outcomes. Non-White race and socioeconomic deprivation have been shown to increase risk of MALE, amputation, and loss of patency after revascularization in CLTI.^11,13,14,16^ From a medical standpoint, DM, dialysis-dependent renal failure, and lack of functional independence have been shown to increase the risk of MALE and early mortality.^33–35^ Worsening WIfI stage is predictive of earlier amputation^26^ Thus, one might have expected significantly worse outcomes in the LPP cohort compared with the traditional clinic cohort.

However, despite these differences in demographics, comorbidities, and disease severity, outcomes within the multidisciplinary cohort were not different from those of patients treated elsewhere in our institution. This remained true even when looking at the most socioeconomically at-risk patient subgroup. These results suggest that socioeconomically deprived patients with many high-risk medical, anatomical, and social characteristics can achieve CLTI outcomes similar to the those of healthier, less deprived patients when cared for by a multidisciplinary center. Semi-structured interviews revealed several key patient-perceived barriers, some of which may be addressed by the structure and design of the multidisciplinary clinic, providing potential explanations for the results we found.

Multidisciplinary clinics have been shown to improve key outcomes in CLTI via retrospective cohort studies, case-control studies, and historically controlled pre-post studies. A recent systematic review and meta-analysis identified reduced mortality, reduced major and minor amputation rates, increased diabetic foot ulcer healing rates, and improved time to ulcer healing.^36^

Most of these studies attribute improvements to the implementation of standardized protocols and the use of evidence-based guidelines within a disease-based clinic.^37–44^ They do not deeply investigate mechanisms by which these clinics attain said outcomes. Other possible mechanisms at play could include prompter or more efficient treatment, improved medical management, provision of consistent high-quality evidence-based wound care by wound specialists, more rigorous follow-up, or improved compliance with lower missed appointment rates. Moreover, there is no research on the role multidisciplinary clinics may have in addressing socioeconomic determinants of health.

This study sets the stage for further investigation into how multidisciplinary clinics may improve outcomes. Our multivariable analysis identified typical risk factors for poor outcomes in CLTI but did not identify clinic type itself as a predictive variable. We wonder if more granular clinic structural elements that were not captured in the present study may be contributing to the normalization of outcomes found within the high-risk LPP cohort. It may also be possible that the very high prevalence of advanced CLTI in our LPP cohort obscured any clinic type effect in the proportional hazards analysis. Subsequent investigations into the previously mentioned proposed mechanisms are needed to understand how multidisciplinary clinics improve outcomes and ultimately design clinic structures that directly contribute to better care. We propose that improved access to care may be one reason that multidisciplinary clinics improve outcomes, and we further believe that this enhanced access may be most beneficial for the most deprived patients with CLTI. Qualitative interviews provided useful insight into this proposition.

Transportation was the most frequently reported barrier. The multidisciplinary clinic addresses some of this barrier with its proximity to a large population of patients with CLTI and comorbid conditions; LPP patients lived significantly closer to the LPP clinic than traditional patients did to their vascular clinics. Furthermore, these transportation restrictions are compounded by the patient-perceived barrier of high visit frequency. The multidisciplinary clinic’s ability to conduct several appointments at one visit at a location that is close to patient homes may therefore address both the transportation and high visit frequency barriers.

The LPP core services include vascular surgery, podiatry, wound care, and prosthetics. However, postoperative medical management was not optimized for some LPP patients. Specifically, we found postoperative prescriptions for statins and P2Y12 inhibitors were less common in the LPP group than the traditional clinic patients. This suggests the importance of including primary care providers or diabetologists more directly. Such inclusion may create a more full-service clinic that expands beyond CLTI while minimizing the burden of additional appointments.

Many interviewed patients reported difficulty understanding their disease, both in terms of management choices and disease severity. CLTI remains a relative mystery among many non-health care professionals, and appropriate communication about the natural course of the disease and optimal treatments is a gap clearly identified by this study. Although our societies and professional organizations attempt to address the population-level issue of decreased awareness of peripheral artery disease, multidisciplinary centers with consistent teams could help to establish rapport and progressively educate members of their local tight-knit communities with high CLTI disease burden and low health care literacy.

The point of establishing rapport is of great interest in mitigating distrust of the health system, a key predictor of adherence to follow-up necessary for patients with vascular disease.^45^ Our study sample’s distrust was centered around both the vascular surgeon themselves and the system as a whole. Establishing rapport with consistent teams found in multidisciplinary CLTI clinics cannot eradicate distrust in the overall health system. However, anecdotally, we, like many others providing CLTI care, have experienced countless instances where LPP patients come to the LPP team first for medical care of any kind in hopes that we can render the care or at least vouch for the trustworthiness of another medical team.

Lastly, racial discordance was identified as a barrier between the patient and their health team. This discordance is not addressed directly by the LPP but highlights the importance of recruiting team members that reflect the ethnic and racial makeup of the local population.

The inability to prove that addressing specific patient-perceived barriers improves outcomes is the greatest limitation of the current work. Future work is necessary to better delineate the associations between individual barriers, specific clinic structural elements, and outcomes within CLTI. For example, we did identify that clinic type might be associated with improved patency in the most deprived patients. Could this reflect more vigorous follow-up protocols? Does proximity to patients result in better surveillance compliance and therefore better patency? Many factors are known to influence outcomes, such as time from presentation to intervention, provision of evidence-based wound care with biweekly visits and debridements, and proper medical management.^1,12,24,39,46–48^ We hope to directly study these factors and their contribution to outcomes in our patient population.

An additional limitation is that all interviewed patients were treated at traditional clinics. Because of faculty turnover and changing responsibilities, LPP operations were temporarily suspended before and during the time interviews were performed. Therefore, patients who received treatment at the LPP did not meet study criteria to be interviewed (ie, intervention within the past 6 months) and were not included in an effort to minimize recall bias. Lastly, all patients in the LPP cohort were treated by a single surgeon. Although this provided the advantage of consistency that potentially addressed some patient-perceived barriers, it also opens our results to significant selection and practice bias. For example, the rate of open and endovascular interventions was different between clinic types.

Note that we do not advocate for concentration of all CLTI care in a smaller number of multidisciplinary centers. Indeed, our data shows no difference in outcomes between the LPP and our traditional clinics. The majority of socioeconomically deprived patients are currently treated at low-volume centers.^11^ A policy of migrating care to a small number of centers housed within major medical centers may decrease access and amplify effects of geographic maldistribution. Instead, a focus on building clinics with essential co-located services, such as vascular surgery and podiatry, within communities with a high burden of CLTI, can address multiple patient-perceived barriers and is likely to be a high-impact intervention.^49^

## CONCLUSIONS

We present a sicker and more socioeconomically deprived cohort of patients with CLTI who were treated at a multidisciplinary LPP and had outcomes that were comparable to those of relatively healthier, less deprived patients with CLTI treated elsewhere in our institution. The multidisciplinary clinic’s proximity to patients, ability to conduct multiple appointments at a single visit, and consistent health care team may have mitigated some barriers to care identified by patients in qualitative interviews. Including these elements in multidisciplinary clinics and placing such clinics in areas of need may help reduce disparities in CLTI. Future work directly quantifying relationships between specific clinic structural features and outcomes may aid future clinic design.

## Supplementary Material

1

## Figures and Tables

**Table I.** Characteristics of patients with chronic limb-threatening ischemia (*CLTI*) that underwent index revascularization are shown, stratified by clinic type

Characteristic	Multidisciplinary LPP	Traditional clinic	*P* value
Age, years	65.12 ± 10.98	66.03 ± 11.06	.3066
Male	122 (63.54)	493 (62.33)	.8188
Non-White race	84 (43.75)	217 (27.43)	<.0001^a^
Hispanic/Latino	0 (0)	11 (1.4)	.2091
Coronary artery disease	72 (37.50)	279 (35.27)	.6212
Congestive heart failure	56 (29.17)	182 (23.01)	.0905
Chronic obstructive pulmonary disease	51 (26.56)	228 (28.82)	.5931
DM	158 (82.29)	484 (61.19)	<.0001^a^
Dialysis-dependent	56 (29.17)	106 (13.40)	<.0001^a^
Hypertension	175 (91.15)	714 (90.27)	.814
Ever smoker	150 (78.13)	654 (82.68)	.1729
Insurance			.2103
Medicaid or self-pay	29 (15.10)	153 (19.34)	
Commercial, Medicare, or Military	163 (84.90)	638 (80.66)	
Residence in nursing home	26 (13.54)	62 (7.84)	.0192^a^
Ambulatory	162 (84.38)	700 (88.83)	.1145
Antiplatelet therapy			
Preoperative P2Y12 inhibitor	65 (33.85)	269 (34.01)	.9999
Postoperative P2Y12 inhibitor	122 (63.54)	565 (71.70)	.0335^a^
Preoperative aspirin	145 (75.52)	573 (72.44)	.4399
Postoperative aspirin	154 (80.21)	611 (77.54)	.4811
Preoperative P2Y12 inhibitor or aspirin	150 (78.13)	644 (81.42)	.3493
Postoperative P2Y12 inhibitor or aspirin	183 (95.31)	751 (95.30)	.9999
Preoperative statin	134 (69.79)	599 (75.82)	.1030
Postoperative statin	141 (73.44)	638 (80.96)	.0267^a^
ADI			
Median^b^	71 (56-92)	72 (54-88)	.0499^a^
ADI >90	52 (29.38)	137 (19.54)	.0061^a^
Required interpreter	4 (2.08)	6 (0.76)	.2149
Distance from clinic, miles^b^	6.73 (3.83-23.34)	28.84 (8.42-54.52)	.0120^a^
Level of disease^a^			
Aortoiliac	24 (12.50)	206 (26.04)	.0001^a^
Femoropopliteal	151 (78.65)	578 (73.07)	.136
Tibial	86 (44.79)	269 (34.01)	.0068^a^
Multilevel disease	70 (36.46)	246 (31.10)	.1803
Index intervention			.0059^a^
Endovascular	157 (81.77)	567 (71.68)	
Bypass	35 (18.23)	224 (28.32)	
WIfI risk of amputation			<.0001^a^
Stage 1	39 (20.97)	144 (21.95)	
Stage 2	33 (17.74)	238 (36.28)	
Stage 3	57 (30.65)	140 (21.34)	
Stage 4	57 (30.65)	134 (20.43)	
TASC			.2629
Type A	30 (15.63)	158 (20.05)	
Type B	42 (21.88)	199 (25.25)	
Type C	59 (30.73)	215 (27.28)	
Type D	61 (31.77)	216 (27.41)	

*ADI*, Area Deprivation Index; *DM*, diabetes mellitus; *LPP*, Limb Preservation Program; *TASC*, TransAtlantic Inter-Society Consensus; *WIfI*, Wound, Ischemia, and foot Infection.

Data are presented as number (%), median (interquartile range), or mean ± standard deviation.

Patients with unknown characteristics were excluded from applicable denominators.

a *P* < .05.

b Failed Shapiro-Wilk test of normality; therefore, non-parametric Mood Median test was used.

**Table II.** Univariable and multivariable Cox proportional hazards analysis of death

Characteristic	Univariable HR	*P* value	Multivariable HR	*P* value
Care at traditional clinic Site	0.89 (0.71-1.12)	.327	1.11 (0.84-1.46)	.467
Age, years	1.03 (1.02-1.04)	<.001^a^	1.03 (1.02-1.04)	<.001^a^
Sex	1.14 (0.92-1.40)	.227	1.20 (0.93-1.54)	.160
Non-White race	1.02 (0.82-1.26)	.891	0.93 (0.70-1.24)	.625
Hispanic or Latino	0.81 (0.30-2.18)	.682	–	–
Coronary artery disease	1.57 (1.28-1.92)	<.001^a^	1.13 (0.87-1.47)	.361
Congestive heart failure	2.99 (2.41-3.7)	<.001^a^	1.96 (1.48-2.61)	<.001
DM	1.35 (1.09-1.68)	.006^a^	1.01 (0.77-1.33)	.954
Dialysis-dependent	2.65 (2.12-3.33)	<.001^a^	2.22 (1.62-3.03)	<.001^a^
Hypertension	2.69 (1.71-4.21)	<.001^a^	1.37 (0.82-2.28)	.229
Ever smoker	0.94 (0.73-1.22)	.643	–	–
Chronic obstructive pulmonary disease	1.68 (1.35-2.07)	<.001^a^	1.47 (1.14-1.90)	.003^a^
Commercial, Medicare, or Military insurance	1.26 (0.96-1.65)	.097	1.00 (0.66-1.53)	.981
Residence in nursing home	1.82 (1.34-2.46)	<.001^a^	0.79 (0.55-1.14)	.203
Ambulatory	0.58 (0.44-0.77)	<.001^a^	1.12 (0.82-1.52)	.475
Postoperative statin	1.32 (1.03-1.69)	.031	1.00 (0.66-1.53)	.981
Postoperative antiplatelet agent	1.24 (0.77-2.00)	.367	–	–
ADI >90	0.94 (0.72-1.23)	.671	1.02 (0.75-1.39)	.884
Distance from clinic, miles	1.00 (1.00-1.00)	.272	–	–
Aortoiliac disease	0.91 (0.71-1.16)	.429	–	–
Femoropopliteal disease	0.99 (0.79-1.25)	.939	–	–
Tibial disease	1.02 (0.83-1.26)	.847	–	–
Multilevel disease	1.07 (0.87-1.32)	.527	–	–
Index intervention				
Endovascular	Ref.	–	–	–
Open bypass	0.71 (0.56-0.90)	.005^a^	0.86 (0.64-1.16)	.321
WIfI risk of amputation	1.22 (1.1-1.36)	<.001^a^	1.13 (1.01-1.27)	.045
TASC	1.03 (0.94-1.13)	.493	–	–

*ADI*, Area Deprivation Index; *CI*, Confidence interval; *DM*, diabetes mellitus; *HR*, hazard ratio; *LPP*, Limb Preservation Program; *Ref*, reference; *TASC*, TransAtlantic Inter-Society Consensus; *WIfI*, Wound, Ischemia, and foot Infection.

HRs and 95% CIs from Cox proportional hazards analysis are shown for death in the entire cohort. Reference rows are noted. Variables with *P* < .2 on univariable analysis, along with clinic type, index intervention, age, race, sex, and ADI >90, were included in the multivariable analysis.

a Characteristics with a *P* < .05 on univariable or multivariable analysis.

**Table III.** Univariable and multivariable Cox proportional hazards analysis of major adverse limb events (*MALE*)

Characteristic	Univariable HR	*P* value	Multivariable HR	*P* value
Care at traditional clinic site	1.00 (0.79-1.28)	.987	1.17 (0.88-1.55)	.280
Age, years	0.99 (0.98-1.00)	.083	0.99 (0.98-1.01)	.348
Sex	1.40 (1.13-1.73)	.002^a^	1.42 (1.11-1.82)	.006^a^
Non-White race	1.15 (0.93-1.42)	.192	1.26 (0.97-1.63)	.081
Hispanic or Latino	1.19 (0.49-2.89)	.694	–	–
Coronary artery disease	0.88 (0.71-1.10)	.262	–	–
Congestive heart failure	0.97 (0.76-1.24)	.809	–	–
DM	1.12 (0.91-1.38)	.298	–	–
Dialysis-dependent	1.46 (1.13-1.88)	.004^a^	1.31 (0.95-1.81)	.095
Hypertension	1.08 (0.78-1.51)	.641	–	–
Ever smoker	0.99 (0.76-1.29)	.930	–	–
Commercial, Medicare, or Military insurance	0.99 (0.77-1.29)	.959	–	–
Residence in nursing home	0.84 (0.57-1.23)	.362	–	–
Ambulatory	0.86 (0.63-1.18)	.356	–	–
Postoperative statin	1.15 (0.90-1.47)	.269	–	–
Postoperative antiplatelet agent	0.99 (0.63-1.57)	.974	–	–
ADI >90	0.99 (0.77-1.29)	.968	0.96 (0.72-1.28)	.764
Distance from clinic, miles	1.00 (1.00-1.00)	.878	–	–
Aortoiliac disease	0.89 (0.70-1.13)	.331	–	–
Femoropopliteal disease	1.29 (1.01-1.65)	.044^a^	1.24 (0.90-1.70)	.193
Tibial disease	1.04 (0.84-1.28)	.746	–	–
Multilevel disease	1.17 (0.95-1.44)	.151	0.99 (0.75-1.32)	.961
Index intervention				
Endovascular	Ref.	–	–	–
Open bypass	1.08 (0.87-1.35)	.471	0.89 (0.66-1.2)	.459
WIfI risk of amputation	1.10 (0.99-1.21)	.076	1.09 (0.98-1.22)	.127
TASC	1.10 (1.00-1.21)	.049^a^	1.10 (0.98-1.24)	.098

*ADI*, Area Deprivation Index; *CI*, Confidence interval; *DM*, diabetes mellitus; *HR*, hazard ratio; *LPP*, Limb Preservation Program; *Ref*, reference; *TASC*, TransAtlantic Inter-Society Consensus; *WIfI*, Wound, Ischemia, and foot Infection.

HRs and 95% CIs from Cox proportional hazards analysis are shown for MALE in the entire cohort. Reference rows are noted. Variables with *P* < .2 on univariable analysis, along with clinic type, index intervention, age, race, sex, and ADI >90, were included in the multivariable analysis.

a Characteristics with a *P* < .05 on univariable or multivariable analysis.

**Table IV.** Themes and illustrative quotations

Theme of barriers	Illustrative quotations
Difficulty understanding disease	“The only thing that’s really confusing to me is that I don’t really know how serious my condition is. I don’t know if I’m getting ready to have a heart attack or if I’m going to die in the next two months.” (Patient 4) “They didn’t do nothing about my foot. I’m not asking to cut it off, but they could have at least fixed it when I was there. They even put me out with a sedative or something. I just don’t understand why they couldn’t do something when I was there.” (Patient 8)
High visit frequency	“I got a diabetes, urology, [vascular], heart failure, and transplant cardiology doctor that I go see. And, then they have other people and it just, you know, it just become a day-to-day job” (Patient 4) “They (vascular, podiatry, endocrinology physicians) have appointments at different times on different days. That makes it really difficult.” (Patient 6)
Transportation barriers	“[My insurance] scheduled a car, but then they left me hanging. 10 minutes before pick up [my insurance] notified me by text and said we don’t have a driver available.” (Patient 1) “I have an eye disease too (from diabetes mellitus), so I’m partially blind. And so getting somebody to bring me is not the easiest.” (Patient 9)
Distrust of the health system	“As far as I’m concerned, the guy (vascular surgeon) was just cocky, young and cocky. He didn’t have his [stuff] together and no Plan B and things didn’t work out like he thought.” (Patient 2) “I’m not happy at all and I’m never going back to anyone down there (the university health system). I’m still getting over being at that hospital and I’ll deal with this on my own… I’m through with this. [Doctors] have screwed me all my life. I’ve lived long enough if this is the worst that happens to me I can deal with it on my own.” (Patient 8)
Patient-physician racial discordance	“They made everything crystal clear, but I couldn’t understand what some of them were saying [due to] their ethnic language.” (Patient 5)

Themes from qualitative interviews, as well as illustrative quotations for each theme are shown. Patient labels are shown to identify which patient the quotes came from.

## ARTICLE HIGHLIGHTS

- **Type of Research:** Explanatory sequential mixed-methods study including a retrospective quantitative component and a qualitative component
- **Key Findings:** The 192 limbs from highly socioeconomically deprived, high-risk patients with chronic limb-threatening ischemia (CLTI) treated at a multidisciplinary clinic had comparable outcomes to the 791 limbs from relatively less deprived, lower-risk patients with CLTI treated elsewhere in the institution. Characteristics of the multidisciplinary clinic may have addressed barriers to care in this population.
- **Take Home Message:** Multidisciplinary clinics may offer socioeconomically deprived patients with CLTI outcomes similar to those of non-deprived patients via mitigation of certain barriers to care.
