# Supplementary material for: How multidisciplinary clinics may mitigate socioeconomic barriers to care for chronic limb-threatening ischemia
Source: J Vasc Surg. Author manuscript; Available in PMC 2024 Nov 25. (PMC11587171; doi:10.1016/j.jvs.2024.05.033)
Supplement: 1 [file NIHMS2034623-supplement-1.pdf]

Supplementary Table I (online only). Univariable and multivariable Cox proportional hazards analysis of patency loss

Characteristic	Univariable HR	*P* value	Multivariable HR	*P* value
Care at traditional clinic site	1.12 (0.87-1.44)	.369	1.24 (0.93-1.64)	.139
Age, years	1.00 (0.99-1.01)	.544	1.00 (0.99-1.01)	.705
Sex	1.17 (0.95-1.44)	.149	1.19 (0.95-1.49)	.134
Race	0.89 (0.71-1.11)	.290	0.89 (0.69-1.15)	.379
Hispanic or Latino	0.84 (0.31-2.24)	.723	–	–
Coronary artery disease	0.91 (0.73-1.13)	.398	–	–
Congestive heart failure	1.08 (0.83-1.40)	.570	–	–
DM	1.00 (0.81-1.24)	.968	–	–
Dialysis-dependent	1.43 (1.09-1.87)	.010[a]	1.57 (1.15-2.14)	.005[a]
Hypertension	1.43 (1.00-2.04)	.048	1.26 (0.85-1.86)	.244
Ever smoker	1.25 (0.94-1.67)	.126	1.29 (0.95-1.76)	.105
Commercial, Medicare, or Military insurance	1.15 (0.88-1.51)	.304	–	–
Residence in nursing home	0.94 (0.63-1.41)	.779	–	–
Ambulatory	1.06 (0.75-1.51)	.741	–	–
Postoperative statin	1.10 (0.86-1.41)	.448	–	–
Postoperative antiplatelet agent	0.78 (0.51-1.21)	.270	–	–
ADI >90	0.94 (0.72-1.23)	.663	1 (0.75-1.33)	.990
Distance from clinic, miles	1.00 (1.00-1.00)	.908	–	–
Aortoiliac disease	0.94 (0.74-1.20)	.640	–	–
Femoropopliteal disease	1.27 (0.99-1.63)	.059	1.22 (0.90-1.64)	.204
Tibial disease	1.06 (0.86-1.31)	.565	–	–
Multilevel disease	1.23 (0.99-1.52)	.062	1.12 (0.86-1.45)	.417
Index intervention				
Endovascular	Ref.	–	–	–
Open bypass	1.26 (1.01-1.57)	.037[a]	1.1 (0.84-1.44)	.498
WIfI risk of amputation	1.06 (0.95-1.18)	.279	–	–
TASC	1.09 (1.00-1.20)	.063	1.09 (0.97-1.21)	.132

ADI, Area Deprivation Index; *CI*, Confidence interval; *DM*, diabetes mellitus; *HR*, hazard ratio; *LPP*, Limb Preservation Program; *Ref*, reference; *TASC*, TransAtlantic Inter-Society Consensus; *WIfI*, Wound, Ischemia, and foot Infection.
HRs and 95% CIs from Cox proportional hazards analysis are shown for patency loss in the entire cohort. Reference rows are noted. Variables with $P < .2$ on univariable analysis, along with clinic type, index intervention, age, race, sex, and ADI >90, were included in the multivariable analysis.
[a]Characteristics with a $P < .05$ on univariable or multivariable analysis.

Supplementary Table II (online only). Complete list of indexed phrases

Difficulty understanding disease	High visit frequency	Transportation barriers	Distrust of the health system	Patient-physician racial discordance
"I didn't have a primary care doctor at the time (of the patient's osteomyelitis). I just was between jobs and [my toe] got infected. I thought [antibiotics] would be enough." (Patient 1)<br>"As far as the pain and the healing process and how long it takes and stuff, I thought it was a simple snip snip, in and out, and off you go. And man, that was not the case." (Patient 2)<br>"The only thing that's really confusing to me is that I don't really know how serious my condition is. I don't know if I'm getting ready to have a heart attack or if I'm going to die in the next two months." (Patient 4)<br>"There are words in that After Visit Summary or something like that that I can't even pronounce. I don't know exactly what they mean or anything. I usually don't have any questions about it until after I get home. Then I get online and then I search for the definitions of the terms or some of the medications. So I don't know, it's just really confusing at times." (Patient 4)<br>"They didn't do nothing about my foot. I'm not asking to cut it off, but they could have at least fixed it when I was there. They even put me out with a sedative or something. I just don't understand why they couldn't do something when I was there." (Patient 8)<br>"Right now [my legs] are feeling pretty good. But I'm just so nervous that it's going to come back. I've had so much pain for the past like four years." (Patient 9)<br>"We went for an angio and when the surgeon had to check the angio they said we had to do surgery. I don't understand why we couldn't have gone for the bypass before the angiogram. It didn't make sense why the bypass had to be a last resort- it should have been possible from the start." (Patient 10)	"Every time I see a doctor or a specialist or something like that, they prescribe a medication, so the medication list got longer. And I have to use my global thing to get the right directions to go to the office or whatever it is. So it's pretty much a job." (Patient 4)<br>"I got a diabetes, urology, [vascular], heart failure, and transplant cardiology doctor that I go see. And, then they have other people and it just, you know, it just become a day-to-day job" (Patient 4)<br>"I would prefer [vascular and wound care] to be at the same location so I could just leave my house and go to one location. But, the thing about OSU is one that deals with cardiovascular or another back up in Upper Arlington does podiatry." (Patient 4)<br>"They (vascular, podiatry, endocrinology physicians) have appointments at different times on different days. That makes it really difficult." (Patient 6)<br>"I prefer [vascular surgery and podiatry appointments] been at the same time so I didn't have to drive as much." (Patient 8)<br>"My sons had to take turns taking time off of work to drive and help translate… It got really hard these last few months because of so many different appointments." (Patient 10)	"[My insurance] scheduled a car, but then they left me hanging. 10 minutes before pick up [my insurance] notified me by text and said we don't have a driver available." (Patient 1)<br>"I have to get a ride because I can't drive without a car. I have some friends that were going to take me and wouldn't because it was a [long ride]. They didn't want to deal with it." (Patient 2)<br>"My office visits have been at [where] I prefer to have them, but we still get a two hour drive." (Patient 3)<br>"I was going to have surgery and my insurance company was supposed to provide transportation for me, but the guy never did come or anything…I ended up having to catch a cab and I actually lost some money." (Patient 4)<br>"It's a long 3-hour drive, but I do have a nephew who lives up there thankfully whose house I can stay at." (Patient 5)<br>"I have no transportation. You know, the last car I had, it was when I was working and I was involved in a really, really bad crash. It totaled the car…But I haven't driven since then because I didn't have a car… And now, I have to rely on other people." (Patient 6)<br>"[Vascular surgeon] told me that that they really want to do a CT scan or whatever you want to call it… I can't do that. I can't get there." (Patient 6)<br>"I had to drive 2 hours… my daughter had to take off work every three weeks for my appointments" (Patient 8)<br>"I have an eye disease too (from diabetes mellitus), so I'm partially blind. And so getting somebody to bring me is not the easiest." (Patient 9)	"The way they approach it at the main hospital is more arrogant. It's not as friendly. And you feel like they're all arrogant, like you feel like they're above you." (Patient 1)<br>"As far as I'm concerned, the guy (vascular surgeon) was just cocky, young and cocky. He didn't have his [stuff] together and no Plan B and things didn't work out like he thought." (Patient 2)<br>One time I said "Wow, I must be their cash cow" (in reference to vascular surgeon) or something because I look at some of the charges for some of these things. I can see [the charges] and some of them are astronomical" (Patient 4)<br>"My hemoglobin was low. They had to give me some medicine to get that hemoglobin up. You know, they never said what they gave me or anything that was never explained. But I believe they gave me the stuff that I was allergic to because I got gout and broke out in a serious rash on my arms and my neck. And it's on you guys." (Patient 6)<br>"I'm not happy at all and I'm never going back to anyone down there (the university health system). I'm still getting over being at that hospital and I'll deal with this on my own… I'm through with this. [Doctors] have screwed me all my life. I've lived long enough if this is the worst that happens to me I can deal with it on my own." (Patient 8)	"Some of the nurses were training from South Africa where I couldn't understand their dialects." (Patient 1)<br>"They made everything crystal clear, but I couldn't understand what some of them were saying [due to] their ethnic language." (Patient 5)

The complete list of indexed phrases are shown along with the theme to which they were assigned and the patient they came from.
